# Delayed by Design: Role of Suboptimal Signal Peptidase Processing of Viral Structural Protein Precursors in Flaviviridae Virus Assembly

**DOI:** 10.3390/v12101090

**Published:** 2020-09-26

**Authors:** Nabeel Alzahrani, Ming-Jhan Wu, Saravanabalaji Shanmugam, MinKyung Yi

**Affiliations:** Department of Microbiology and Immunology, University of Texas Medical Branch at Galveston, 301 University Boulevard, Galveston, TX 77555-1019, USA; naalzahr@utmb.edu (N.A.); miwu@utmb.edu (M.-J.W.); shsarava@utmb.edu (S.S.)

**Keywords:** Flaviviridae, flavivirus, hepacivirus, HCV, pestivirus, signal peptidase, delayed processing, nucleocapsid, virus assembly

## Abstract

The Flaviviridae virus family is classified into four different genera, including flavivirus, hepacivirus, pegivirus, and pestivirus, which cause significant morbidity and mortality in humans and other mammals, including ruminants and pigs. These are enveloped, single-stranded RNA viruses sharing a similar genome organization and replication scheme with certain unique features that differentiate them. All viruses in this family express a single polyprotein that encodes structural and nonstructural proteins at the N- and C-terminal regions, respectively. In general, the host signal peptidase cleaves the structural protein junction sites, while virus-encoded proteases process the nonstructural polyprotein region. It is known that signal peptidase processing is a rapid, co-translational event. Interestingly, certain signal peptidase processing site(s) in different Flaviviridae viral structural protein precursors display suboptimal cleavage kinetics. This review focuses on the recent progress regarding the Flaviviridae virus genus-specific mechanisms to downregulate signal peptidase-mediated processing at particular viral polyprotein junction sites and the role of delayed processing at these sites in infectious virus particle assembly.

## 1. Introduction

The Flaviviridae family represents a group of enveloped, single-stranded, positive-sense RNA viruses belonging to four different genera including flavivirus, hepacivirus, pegivirus, and pestivirus. Most viruses belonging to the flavivirus genus are transmitted by infected arthropod vectors, hence called arboviruses, and several members in this genus, including Dengue virus (DENV), Japanese encephalitis virus (JEV), Tick-borne encephalitis virus (TBEV), West Nile virus (WNV), and Zika virus (ZIKV), are associated with widespread morbidity and mortality in the human population [1]. Within the hepacivirus genus, the hepatitis C virus (HCV) is the only blood-borne human pathogen, chronically infecting an estimated 71.1 million people, and causes severe liver diseases, including chronic hepatitis, cirrhosis, and hepatocellular carcinoma [2]. Human pegivirus, previously known as hepatitis G virus (HGV), is commonly detected in healthy blood donors but has not been associated with any disease [3]. Pestiviruses, including Bovine viral diarrhea virus (BVDV), Border disease virus (BDV), and Classical swine fever virus (CSFV), cause substantial economic losses by infecting pigs and ruminants [4].

The positive-sense RNA genome of Flaviviridae family members contains a single open reading frame (ORF) flanked by the 5′ and 3′ non translated region (NTR). The ORF is translated to a single polyprotein, either via cap-dependent translation (flavivirus) or internal ribosome entry site (IRES)-mediated translation (hepacivirus, pegivirus, and pestivirus) [5,6,7]. The viral polyprotein is processed by the host and viral proteases, co- and -post-translationally, into 9 to 12 mature proteins, consisting of the structural proteins, including Capsid or Core (C) and envelope (E) glycoproteins, and nonstructural (NS) proteins [1,4,7,8].

**Figure 1:**
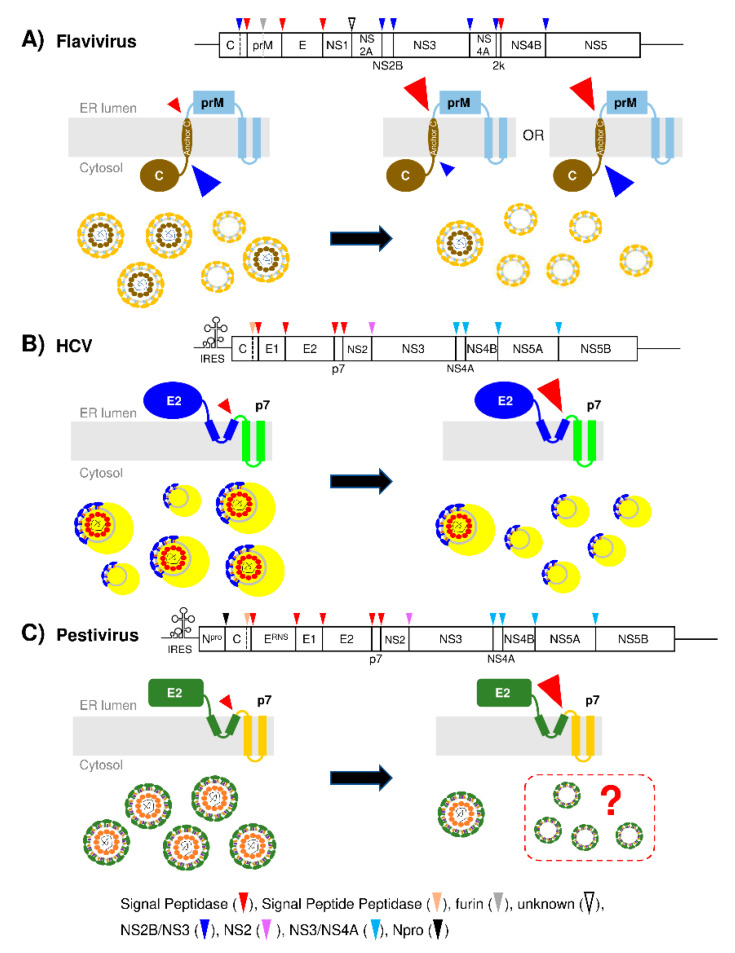
Flaviviridae virus genome organization and delayed signal peptidase cleavage sites involved in virus assembly. (**A**) The top panel shows the flavivirus genome organization marked with host- and virus-derived protease processing locations. The middle panel shows the C–prM processing by signal peptidase (red arrowhead) and NS2B/NS3 protease (blue arrowhead). The bottom panel shows the relative production of nucleocapsid-containing infectious particles and nucleocapsid-free subviral particles depending on the efficiency of signal peptidase-mediated processing of C–prM junction. The size of the arrowhead indicates the relative cleavage efficiency, with big and small sizes corresponding to efficient and inefficient processing, respectively, assessed by the steady-state levels of cleaved products compared to the precursor(s). Infectious virus particles and relatively small, nucleocapsid-free subviral particles (SVPs) are shown. The relative numbers of virions and SVPs in this figure do not represent their biological proportion. The left side of the figure shows natural, delayed processing by signal peptidase (SPase) at the indicated processing site allowing infectious virus production, and the right side of the figure shows modified conditions altering either SPase- or viral protease-mediated cleavage efficiency, leading to reduced virus production while still allowing nucleocapsid-free SVPs production. (**B**) HCV genome organization and the role of delayed E2–p7 processing in nucleocapsid-containing infectious lipoviroparticles (LVPs) production. The yellow portion of LVPs represents lipoprotein components. (**C**) Pestivirus genome organization and tentative role of delayed E2–p7 processing in infectious pestivirus production. Nucleocapsid-free SVPs have not been described for pestivirus and whether enhanced E2–p7 processing may lead to the production of this type of SVP is unknown, as indicated with a question mark within the dashed line box.

In general, with some exceptions, Flaviviridae virus-encoded proteases carry out the NS protein processing and host signal peptidase (SPase) cleaves most of the structural protein junction sites (Figure 1). In principle, SPase-mediated cleavage is expected to occur in a rapid, co-translational manner [9,10]. However, among the three SPase-dependent cleavage sites within the C–prM–E–NS1 region of flavivirus, C–prM cleavage is delayed [11,12,13] (Figure 1A). Likewise, among the four SPase target sites within the C–E1–E2–p7–NS2 region of HCV (hepacivirus), E2–p7 and, to a lesser extent, p7–NS2 processings are delayed and/or incomplete [14,15,16] (Figure 1B). In the case of pestivirus, among five SPase target sites within the C–E^rns^–E1–E2–p7–NS2 region, E^rns^–E1 and E2–p7 processing are delayed and/or incomplete [17,18,19] (Figure 1C). Recent studies indicate that altering the SPase-mediated processing efficiency at these “delayed” cleavage sites reduced virus assembly and/or release, suggesting that maintaining the “optimal inefficiency” of SPase-mediated cleavage at these sites is essential for viral propagation [11,16,18,20,21,22,23]. By discussing recent progress, this review aims to provide insight on how different viruses within the Flaviviridae family differentially regulate the host SPase-mediated cleavage at specific junction site(s) within viral structural protein precursors and how the calculated regulation of SPase processing at these sites contributes to their efficient propagation.

## 2. C–prM Cleavage Regulation in Flavivirus Assembly

### 2.1. A Brief Overview of Flavivirus Assembly

Flavivirus NS proteins mediate and regulate viral RNA replication by inducing the ER-derived replication factory called vesicle packets (Vp), which contain a small cluster of double-membrane vesicles (Ve) housing the replication complexes (RC) [1,24,25]. Newly generated plus-sense viral RNAs are subjected to a new round of RNA replication, protein translation, or virus assembly. It was proposed that virion assembly occurs when the newly synthesized genomic RNA, secreted through the Ve pore, interacts with capsid (C) proteins for encapsidation, followed by an envelopment of nucleocapsids as they bud into the adjacent ER membrane harboring PrM and E. This model is supported by visualization of the budding virions from ER sites juxtaposed to the Ve pore for different flaviviruses, including DENV, WNV, and ZIKV [24,25,26,27]. According to flavivirus crystal structure information, the nucleocapsid (NC) consisting of 120 copies of C protein and a single genomic RNA buds into the ER membranes, having 180 copies of prM/E heterodimers forming immature viral particles [28,29]. Maturation of viral particles occurs during their trafficking through the trans-Golgi network upon cleavage of prM into pr and M by host protease furin, before the release of the infectious mature virus into extracellular environment [30]. Until recently, however, it was unclear how flavivirus specifically encapsidates its genomic RNA, since the RNA binding activity of C protein lacks sequence specificity [31]. This mystery was addressed in two recent reports by Xie et al. and Zhang et al., which provided evidence that, for both ZIKV and DENV, NS2A is responsible for recruiting viral RNA to the virion assembly sites by specifically interacting with 3′ NTR of viral RNA [32,33]. These authors also demonstrated that NS2A additionally recruits C–prM-E polyprotein and NS2B/NS3 protease to the virus assembly sites and regulate C–prM cleavage to orchestrate the virion assembly.

### 2.2. Role of Delayed C–prM Cleavage in Flavivirus Assembly

#### 2.2.1. Mechanism of Delayed C–prM Cleavage

Three junction sites within the C–prM-E-NS1 region of flavivirus are processed by SPase in the lumen of the endoplasmic reticulum (ER). Ectopic expression of this region showed that the C–prM junction is processed poorly, unlike the other two junction sites, which are efficiently processed [11,34]. C–prM processing was enhanced by prior NS2B/NS3-mediated cleavage at the cytosolic portion of C protein after the two basic residues preceding the transmembrane domain (anchor C), which also serves as a signal sequence for prM [11,12,20,21,34,35] (see Figure 1A, middle panel, for the SPase and NS2B/NS3 protease cleavage sites located at the cytosolic- and ER luminal-side of anchor C, respectively). The typical signal sequence consists of a positively charged n-region, hydrophobic h-region spanning the membrane, and relatively polar c-region involved in recognition and cleavage by SPase [36]. The anchor C/signal sequence is suboptimal for SPase-mediated cleavage due to the nonpolar nature of the c-region and optimizing this region enhanced the SPase-mediated C–prM cleavage, independent of prior cleavage of C protein by NS2B/NS3 [11,20,21]. The enhanced C–prM cleavage by SPase, however, inhibited virus assembly, indicating that delayed C–prM cleavage is critical for efficient flavivirus assembly [20,21,37]. Of note, in addition to the suboptimal signal sequence, recent findings indicate that NS2A could provide an additional layer of regulation for C–prM cleavage by modulating NS2B/NS3-mediated C protein cleavage efficiency via its interaction with both prM and NS2B/NS3, facilitating NS2B/NS3 recruitment near to its substrate C protein and finetuning the accessibility of the NS2B/NS3 cleavage site at the cytosolic side of anchor C [32,33].

#### 2.2.2. Role of C–prM Cleavage in Flavivirus Assembly

The C protein was considered dispensable for the flavivirus membrane budding process since prM/E expression by themselves, in the absence of any other viral proteins including the C protein, could drive subviral particle (SVP) budding [38]. It was hypothesized that the mature form of the capsid (m-C), generated following internal cleavage of C protein by NS2B/NS3 and thus, lacking the anchor C region, interacts with the viral lipid membrane via its hydrophobic patch and the negatively charged viral RNA via the positively charged surface area [39]. However, recent cryoEM structural analysis of Zika immature particles by Tan et al. indicated that full-length C protein (f-C) likely plays an active role in flaviviral virion formation [28]. According to this report, trimers of mixed f- and m-C protein dimers held together by anchor C domain (or helix α5) interact with transmembrane hairpins of 9 preformed prM/E complexes embedded in the ER membrane, forming an assembly unit, leading to NC-containing viral particle budding [28]. These authors proposed that the presence of some f-C proteins may determine the spacing between the prM/E proteins, leading to correct icosahedral infectious particle conformation [28,40].

It is intriguing to note that enhancing the C–prM cleavage by SPase, thereby uncoupling the sequential cleavage of C–prM, first by NS2B/NS3 and then by SPase, inhibited flavivirus assembly by reducing NC incorporation to the virion [20,21,37] (Figure 1A). However, mechanistic insight for the role of sequential C–prM processing in NC incorporation to the virion remains unclear. Based on the newly illuminated role of f-C protein and NS2A in flavivirus assembly, the following scenario may be envisioned. First, the deliberately inefficient SPase processing would ensure the recruitment of C and prM to the same virus assembly sites in the form of a C–prM precursor via interaction between prM and NS2A [11,32,33,34]. This could explain why enhancing the C–prM cleavage by SPase, regardless of efficient or inefficient internal processing of C by NS2B/NS3, reduced the NC incorporation to the viral particles [20,21,37] (Figure 1A). Meanwhile, E protein recruitment to the virus assembly sites likely occurs along with prM via co-translational heterodimerization between prM and E [41,42]. Second, the recruitment of NS2B/NS3 by NS2A to the same sites will allow its processing of C protein at the cytosolic side of anchor C, generating the m-C protein, and promote the subsequent, SPase-mediated processing of C–prM junction site [11,32,33,34]. Consistent with this possibility, NS2B/NS3-mediated C protein cleavage was proposed to occur in trans rather than in cis during flavivirus replication, since C–prM cleavage, which depends on prior NS2B/NS3 cleavage of the C protein, occurred faster than the time required for translating the downstream NS2B/NS3 after C–prM translation [41]. Third, some C–prM precursors may be processed by SPase first, leading to the generation of f-C protein. Supporting this possibility, augmenting C–prM processing by SPase led to f-C generation even in the presence of NS2B/NS3 [20]. Forth, m-C and f-C protein will interact with the viral genome recruited by NS2A to form the NC [28,32,33]. Fifth, the assembly of NC-containing immature virus particles occurs when the NC buds into the ER membrane enriched with the prM and E proteins. Tan et al. proposed that this process is initiated by the interaction between C proteins and the transmembrane region of the prM and E proteins on the cytosolic side of the ER, forming an assembly unit consisting of trimers of a mixture of f- or m-C protein dimers complexed with 9 preformed prM/E complexes [28,40]. Subsequently, the lateral interaction between the ectodomains of prM/E complexes on the luminal side of the ER drives icosahedral virus particle assembly [28,40]. Sixth, maturation of immature particles into infectious virions occurs during their transport through the secretory pathway mediated by the large conformational arrangement of the E protein and the cleavage of prM to M protein by furin protease [28,30,40,43].

## 3. Role of E2–p7 Cleavage Regulation in HCV Assembly

### 3.1. Overview of HCV Assembly and Release

The HCV NS3-NS5B region flanked with 5′ and 3′-NTR is sufficient to support HCV RNA replication [44,45,46]. Expression of these NS proteins, including the key player NS5A, led to the formation of double membrane vesicles (DMVs) that serve as an HCV replication compartment [47,48,49,50,51], where amplification of HCV RNA occurs [8,52,53]. Then, HCV assembly occurs by hijacking the VLDL assembly/secretion pathway to make a unique form of virus called lipoviroparticles (LVPs), which are composed of lipoprotein-like lipids, several apolipoproteins, including apoB, apoE, apoCI, and apoCIII, in addition to viral RNA, Core, E1, and E2 [54,55,56,57,58,59,60]. While the exact mechanism of HCV assembly is still incompletely defined, critical steps in the viral assembly process have emerged. HCV capsid protein (Core) associates with the cytoplasmic lipid droplets (cLDs) upon the signal peptide peptidase (SPP)-mediated intramembrane cleavage of the E1 signal peptide region at the C-terminus of Core [61,62]. Conflicting conclusions were reported regarding the requirement of the prior processing of the Core–E1 junction by SPase on SPP-mediated Core cleavage [61,63,64,65,66]. By employing multiple mutations, cell types, and HCV genotypes used in earlier studies, in addition to the HCV infection system, Pène et al. clarified this controversy and established the sequential processing of HCV Core, first by SPase to release the Core from the polyprotein, followed by SPP to generate mature Core [62]. The cLD-associated mature Core recruits NS5A, presumably loaded with HCV RNA, to the same cLDs, which would trigger encapsidation [67,68,69]. Next, NS2 recruits envelope proteins E1 and E2 to the ER detergent-resistant membranes (ER-DRM) near the Core-decorated cLD [70,71,72,73,74], which likely trigger envelopment/budding of HCV particles into the ER lumen. NS2 palmitoylation facilitates NS2-mediated E2 recruitment to the virus assembly sites located at the ER-DRM, since inhibiting NS2 palmitoylation reduced ER-DRM localization of NS2 and E2, and colocalization of NS2 with cLD-associated Core [74]. The coordination of HCV RNA replication and virus assembly was captured by the state-of-the-art imaging study by Lee et al., which showed cLD wrapping by the DMV-associated ER membranes and E2 recruitment to these membranes during HCV replication [75]. The physical proximity between the compartments for HCV RNA replication and assembly should facilitate nucleocapsid-containing virion assembly, preceding infectious HCV lipoviroparticle (LVP) formation by acquiring lipoprotein components including the lipids and apolipoproteins, such as ApoB and ApoE [76,77,78,79]. Interestingly, acquiring ApoE and lipids in the HCV virions could occur intracellularly during HCV egress following nucleocapsid formation and envelopment [80] or after viral secretion to the extracellular space [81,82,83]. For additional insight regarding HCV assembly and maturation, readers should refer to previous reviews [8,58,84].

### 3.2. Role of Delayed E2–p7 Processing in HCVs Assembly

#### 3.2.1. Mechanism of Delayed E2–p7–NS2 Cleavage

Four junction sites within the HCV C–E1-E2–p7–NS2 region are processed by SPase. Among them, processing at the E2–p7 and, to a lesser extent, p7–NS2 junction sites is delayed, leading to the detection of E2–p7 or E2–p7–NS2 precursors in an ectopic protein expression system [15,16,85]. The signal peptide sequences located at the N-terminal of these cleavage sites showed no defect when fused to a reporter protein, indicating that suboptimal cleavage at these sites is not due to defective signal sequence [14], different from that of the flavivirus C–prM cleavage delay as described above. Instead, Carrère-Kremer et al. showed that the N-terminal sequence of p7 and NS2 located at the P’ side of the SPase cleavage site impeded the processing at the preceding cleavage site, potentially by imposing a structural constraint [14]. The N-terminal sequence of p7 additionally regulated the p7–NS2 cleavage by SPase [14,16]. Processing of E2–p7 and p7–NS2 precursors occurs independently, as blocking either one of these two processing sites did not alter the processing at the other site [14]. Interestingly, the E2–p7 precursor was more readily detectable than the p7–NS2 or E2–p7–NS2 precursors during HCV replication. It was shown that ~15% of E2 from HCV genotype 1a (gt1a) H77 strain, and ~40% and ~50% of E2 from two different gt2a strains, J6 and JFH1, respectively, was detected in the E2–p7 precursor form during viral replication [16,23]. The relatively minor levels of p7–NS2 precursors during HCV replication suggest that either this junction site is readily processed or these precursors are short-lived in the context of HCV replication [86].

#### 3.2.2. Role of E2–p7 Processing in HCV Assembly and Release

Consistent with the regulatory role of the p7 N-terminal domain in E2–p7 processing by SPase described above [14], several mutations introduced to this domain in the context of infectious HCV genome either up- or downregulated E2–p7 processing efficiency [23,87]. The mutations that impaired E2–p7 processing often resulted in a lack of HCV assembly while showing no impact on HCV RNA replication and the compensatory mutations, which restore the infectious virus production defect of these mutants, also reversed the E2–p7 processing defect [16,87]. These results indicate that E2–p7 cleavage is critical for HCV particle assembly [87]. Interestingly, the mutations that enhanced E2–p7 processing above wt level also disrupted infectious HCV assembly and release [23]. These results suggest that naturally suboptimal E2–p7 processing is critical for efficient HCV production.

How does delayed E2–p7 processing regulate HCV production? Denolly et al. described that p7 N-terminus mutations that enhanced E2–p7 processing reduced the Core and HCV RNA incorporation to the secreted particles, while enhancing the E1/E2-containing particle secretion, consequently reducing infectious HCV production [23] (Figure 1B). Like flavivirus, HCV E1 and E2 expression alone could drive subviral particle formation [23,88]. Interestingly, ~90% of E2 secreted from gt2a chimera JC1 replicating cells was detectable in the density gradient fractions representing the nucleocapsid-free subviral particles (SVPs) [23], consistent with previous findings by Scholtes et al. which demonstrated the predominance of nucleocapsid-free SVPs over infectious virions in the plasma of hepatitis C patients [89]. These results suggest that HCV nucleocapsid envelopment is an inefficient event. The delayed E2–p7 likely contributes to HCV assembly by regulating the release of p7, which has been implicated in nucleocapsid formation. Gentzsch et al. demonstrated that p7 mutants caused the accumulation of capsid assembly intermediates lacking envelopment and HCV RNA protection, indicating a defect in nucleocapsid formation [90]. Boson et al. showed that p7, in coordination with NS2, regulates HCV Core localization to the ER, which is required for virus assembly, supporting the role of p7 in nucleocapsid assembly [91]. We and others showed that inhibition of E2–p7 processing disrupted NS2-mediated E2 recruitment to the virus assembly sites located at the ER-DRM due to the lack of functional p7, which regulates NS2 recruitment to the ER-DRM [16,92,93]. In other words, the controlled release of p7 due to delayed E2–p7 processing, by enhancing Core and envelope protein recruitment to virus assembly sites, promotes nucleocapsid budding into the envelope protein-containing membranes for virion formation.

P7 is a viroporin, which forms a cation-selective ion channel typically as hexa- or heptameric oligomers [94,95]. Wozniak et al. demonstrated that the ion channel function of p7 reduces the acidification of intracellular compartments, which was critical for infectious virus production, possibly by protecting the nascent virus particles from the acidic environment during maturation/egress [96]. Supporting this notion, either the inhibitor of cellular organelle acidification (Bafilomycin A1) or influenza viroporin M2 could partially restore the defect in HCV infectivity caused by p7 mutations [87,96,97]. However, spontaneous ion channel formation by p7 at the ER membrane could lead to apoptosis induction [98]. Therefore, the additional role of delayed E2–p7 processing, by regulating p7 release, is likely to mitigate this potential cytotoxicity and promote sequential assembly of p7 oligomers for gradual formation of the active ion channels involved in infectious HCV egress, as proposed by Chandler et al. [99].

## 4. Role of Pestivirus E2–p7 Cleavage Regulation in Virus Assembly

### 4.1. Overview of Pestivirus Assembly

Pestivirus replication and assembly mechanisms are poorly defined compared to those of flavivirus or HCV. However, many aspects of pestivirus replication closely resemble those of HCV, despite some differences. Pestivirus genome organization is similar to that of HCV, although pestivirus encodes two extra proteins, including N^pro^ and E^rns^. As in the case of HCV, pestivirus polyprotein translation is mediated by IRES encoded within the 5′-NTR region [6,100,101]. Similar to HCV, the pestivirus NS3-NS5B coding region, flanked by 5′- and 3′-NTR, supported autonomous RNA replication, although pestivirus RNA replication required an additional minimum of 39 nucleotides of the N^pro^ sequence, likely to enhance IRES-dependent translation initiation [102,103,104]. However, unlike HCV, pestivirus replication did not induce obvious membrane rearrangement, potentially suggesting a different mode of viral RNA replication compartment generation [105]. Like HCV, SPP-mediated intramembrane cleavage of Core is involved in the efficient propagation of pestivirus [106,107,108,109]. However, despite being cleaved by SPP, unlike HCV Core, pestivirus Core was mainly associated with the ER, not cLDs [105]. Pestivirus assembly consists of nucleocapsid budding into the ER lumen to form relatively homogenous ~50 nm viral particles [105,110]. While the exact mechanism of the pestivirus assembly process remains unclear, it is likely different from that of HCV, since pestivirus assembly does not involve the hijacking of lipoprotein metabolism. Following assembly in the ER, pestivirus virions are secreted via the constitutive cellular secretory pathway by transferring to the ER-Golgi intermediate compartment, then the Golgi compartment, followed by single-particle exocytosis [105,111,112].

### 4.2. Role of Delayed Pestivirus E2–p7 Processing: An Identical Twin of HCV?

Five junction sites within the pestivirus C–E^rns^–E1–E2–p7–NS2 region are processed by SPase. Among these, E^rns^–E1 and E2–p7 processing were delayed [17,18,19]. The C-terminus of E^rns^ forms an amphiphilic helix that serves as a membrane anchor, SPase cleavage site, and a retention/secretion signal [113]. An atypical SPase cleavage site lacking the usual, membrane-spanning signal sequence could explain the relatively inefficient processing at the E^rns^–E1 junction site. E^rns^ is one of four structural proteins present on the pestivirus virion, which also include Core, E1, and E2, but is also secreted to extracellular space, functioning as a virulence factor based on its RNase activity [110,114,115]. Delayed E^rns^–E1 processing likely regulates the multifunctional roles of E^rns^ by controlling the relative levels of E^rns^ that are destined to secretion and viral incorporation. However, besides this, the specific role of delayed E^rns^–E1 cleavage in pestivirus assembly, if any, remains unclear.

The mechanism responsible for the incomplete processing of pestivirus E2–p7 is unknown. However, similar to HCV, artificial deregulation of pestivirus E2–p7 processing, either by introducing a mutation to the last residue of E2 to abolish SPase-mediated cleavage or complete separation of these two proteins by inserting a stop codon at the end of E2 followed by EMCV IRES between these two proteins, impaired infectious virus production without affecting viral RNA replication [16,18,116]. The identical phenotypic outcomes following modification of E2–p7 processing in pestivirus and HCV suggest that the mechanistic role of delayed E2–p7 processing in pestivirus production may be similar to that of HCV (Figure 1C). To support this notion, like HCV p7, pestivirus p7 is also a viroporin, and introducing amino acid mutations to pestivirus p7 also inhibited infectious virus production without affecting viral RNA replication [116,117]. Thus, delayed pestivirus E2–p7 processing likely regulates the controlled release of p7 to promote the p7-mediated functions in virus assembly as in the case of HCV. However, it is important to note that pestivirus SVPs lacking nucleocapsid have never been described so far and, therefore, further study is necessary to either confirm the above hypothesis or determine the actual, potentially unique, mechanistic role of delayed E2–p7 processing in pestivirus assembly.

## 5. Conclusions

At first glance, the role of delayed SPase-mediated processing at the C–prM junction site in flavivirus and the E2–p7 junction site in HCV seems distinct, since the former cleavage separates the capsid and membrane protein, while the latter cleavage release the envelope protein and viroporin. However, in both cases, the main outcome of delayed processing at these sites seems to facilitate nucleocapsid incorporation to the virions, since enhancing the processing at these sites inhibited the formation of nucleocapsid-containing particles while permitting the formation of subviral particles lacking nucleocapsid [20,21,23] (Figure 1A,B). In pestivirus, the role of delayed E2–p7 processing in viral replication remains undefined other than that this processing is critical for infectious virus assembly [18]. However, considering the similar phenotypic outcomes of altering E2–p7 processing in virus assembly between pestivirus and HCV, pestivirus E2–p7 processing delay potentially has a similar purpose to that of HCV (Figure 1C). Regardless, at least for flavivirus and HCV belonging to the same Flaviviridae family, they share a common strategy involving the purposeful impediment of host SPase-mediated viral structural protein cleavage to facilitate infectious, nucleocapsid-containing particle assembly.
